# Inhibition of Hyaluronan Synthesis Reduces Versican and Fibronectin Levels in Trabecular Meshwork Cells

**DOI:** 10.1371/journal.pone.0048523

**Published:** 2012-11-05

**Authors:** Kate E. Keller, Ying Ying Sun, Janice A. Vranka, Lauren Hayashi, Ted S. Acott

**Affiliations:** The Casey Eye Institute, Oregon Health & Science University, Portland, Oregon, United States of America; University of Regensburg, Germany

## Abstract

Hyaluronan (HA) is a major component of the extracellular matrix (ECM) and is synthesized by three HA synthases (HAS). Similarities between the HAS2 knockout mouse and the hdf mutant mouse, which has a mutation in the versican gene, suggest that HA and versican expression may be linked. In this study, the relationship between HA synthesis and levels of versican, fibronectin and several other ECM components in trabecular meshwork cells from the anterior segment of the eye was investigated. HA synthesis was inhibited using 4-methylumbelliferone (4MU), or reduced by RNAi silencing of each individual HAS gene. Quantitative RT-PCR and immunoblotting demonstrated a reduction in mRNA and protein levels of versican and fibronectin. Hyaluronidase treatment also reduced versican and fibronectin levels. These effects could not be reversed by addition of excess glucose or glucosamine or exogenous HA to the culture medium. CD44, tenascin C and fibrillin-1 mRNA levels were reduced by 4MU treatment, but SPARC and CSPG6 mRNA levels were unaffected. Immunostaining of trabecular meshwork tissue after exposure to 4MU showed an altered localization pattern of HA-binding protein, versican and fibronectin. Reduction of versican by RNAi silencing did not affect HA concentration as assessed by ELISA. Together, these data imply that HA concentration affects synthesis of certain ECM components. Since precise regulation of the trabecular meshwork ECM composition and organization is required to maintain the aqueous humor outflow resistance and intraocular pressure homeostasis in the eye, coordinated coupling of HA levels and several of its ECM binding partners should facilitate this process.

## Introduction

Trabecular meshwork (TM) cells are a specialized endothelial cell type in the anterior segment of the eye. [1] TM cells have two main functions: One is to phagocytose debris present in aqueous humor as it passes through the filter-like tissue of the TM and drains into Schlemm’s canal. [2], [3] The other main function of TM cells is to modulate the flow resistance to aqueous humor in order to maintain intraocular pressure (IOP). [4], [5] The TM cells responsible for IOP homeostasis reside deep within the TM in an area closest to Schlemm’s canal, a region known as the juxtacanalicular (JCT) or cribriform region. Homeostatic adjustments of the outflow resistance are responsible for maintaining IOP levels. At a cellular level, modification of outflow resistance appears to involve cellular recognition of elevated IOP as a stretch/distortion, focal proteinase degradation of the existing extracellular matrix (ECM) and biosynthesis of replacement components. [5], [6], [7] The replacement components differ in the overall amounts and/or contain different modular domains due to altered alternative mRNA splicing. [8] This newly deposited ECM thus alters the resistance to aqueous outflow. Dysregulation of this tightly regulated balance of degradation and synthesis can result in elevated IOP. Persistent elevated IOP is the major risk factor for primary open-angle glaucoma (POAG). [9].

A direct contribution of ECM to the outflow resistance was first evoked based on perfusion of glycosaminoglycan (GAG)–degrading enzymes into ocular anterior segments. For instance, degradation of hyaluronan (HA) by hyaluronidase was found to decrease outflow resistance and consequently increase outflow facility in bovine eyes. [10], [11], [12], [13] HA is a large, negatively charged GAG chain that is synthesized by three related HA synthases called HAS1, HAS2 and HAS3. [14], [15], [16] HAS enzymes alternately add UDP-*N*-acetyl glucuronate (UDP-GlcUA) and UDP-*N*-acetyl-glucosamine (UDP-GlcNAc) to the reducing end of a growing HA chain near the cell surface, where it is extruded directly into the extracellular space. The rate of HA synthesis can be modulated by the cytosolic concentration of UDP-sugar substrates. [17] Each HAS gene synthesizes different amounts and sizes of HA: HAS1 produces small amounts of high molecular weight HA, HAS2 produces high amounts of high MW HA, while HAS3 produces high amounts of low MW HA. [18], [19] HA comprises approximately 20–25% of total GAG chains in the TM and is concentrated in the JCT region of the tissue. [20], [21] This observation, combined with the GAG-degradation outflow experiments, suggests that HA is a significant component of the outflow resistance. Recently, the effects of 4-methylumbelliferone (4MU), an inhibitor of HA synthesis, [22], [23], [24] and RNAi silencing of each individual HAS gene on outflow facility in an ocular perfusion culture model of aqueous humor outflow was reported. [25] Inhibition of HA synthesis by both methods increased outflow resistance in human eyes.

Versican is a large, chondroitin sulfate (CS)-substituted proteoglycan that binds HA through its G1 domain. [26] Alternative splicing of versican results in multiple transcript variants called V0, V1, V2 and V3. The V1 isoform is the predominant transcript expressed by TM cells. [8], [27] Versican appears to play a central role in outflow resistance, conceivably via its ability to organize other ECM components to facilitate and control open flow channels in the JCT. [28] Degradation of versican by the enzyme ADAMTS4 (a disintegrin and metalloproteinase with thrombospondin motifs 4) decreased outflow resistance, but RNAi silencing of versican increased outflow resistance in perfused human eyes. [28], [29] Fibronectin is also a major component of the TM. [30] Fibronectin has many biological functions and can influence cell signaling via regulated interactions with its cell surface receptors, the integrins. Perfusion of the HepII domain of fibronectin decreased outflow resistance in human and monkey eyes, which demonstrates a role for fibronectin in outflow resistance. [31], [32].

Previously, similarities between the HAS2 knockout mouse and the hdf mouse, which has a random insertion mutation in the versican gene, have been described. [33], [34], [35] Both of these mice strains die *in utero* between E9.5 and E10.5 from cardiovascular abnormalities. The mRNA expression of HAS2 and versican overlaps both spatially and temporally in the developing heart in wild-type mice, which has led to the suggestion that HA and versican are linked during morphogenesis. [33] This is probably due to their specific interaction and operation as a functional complex at the cell surface, which can in turn modulate cellular signaling. In this study, we investigate the relationship between HA synthesis and versican and fibronectin levels in TM cells.

## Results

### Effects of 4MU on Versican

**Figure 1:**
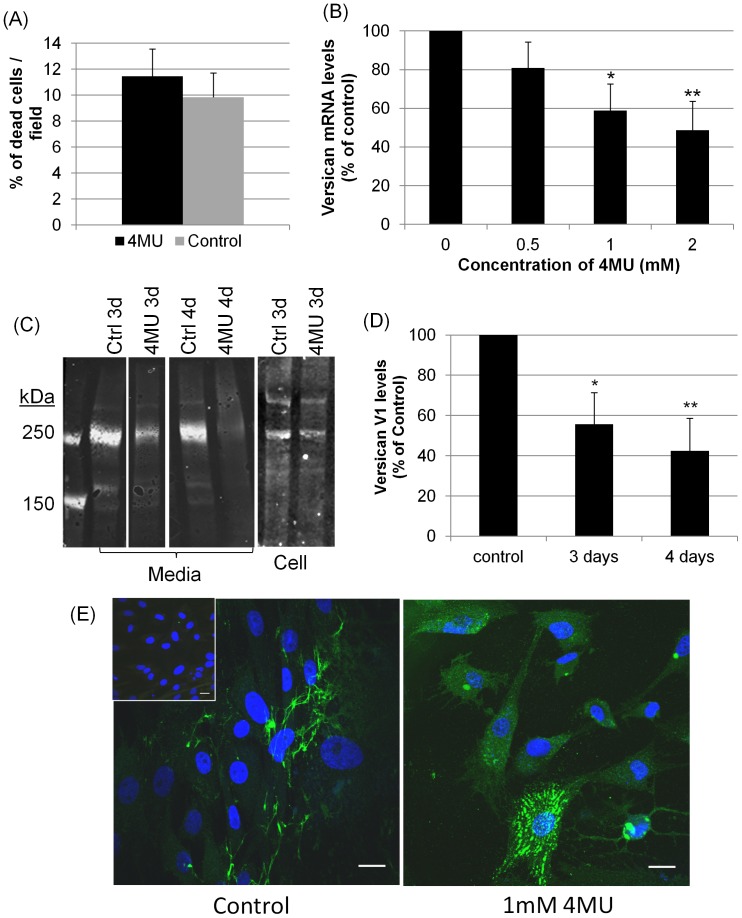
Versican levels in response to 4MU treatment. (A) A cell viability assay showed no significant difference (p = 0.575) in the percentage of dead cells per field between TM cells treated for 5 days with 1 mM 4MU or vehicle control. Error bars are s.e.m; n = 6 fields counted. (B) Versican mRNA levels were assessed by qRT-PCR in TM cells treated with 0, 0.5, 1 and 2 mM 4MU for 24 hours. mRNA levels were normalized to 18S RNA levels and presented as a percentage of the vehicle control. Error bars are the s.e.m.; n = 3; * p = 0.04 and ** p = 0.026 by one-way ANOVA. (C) Western immunoblots of versican in conditioned media and RIPA lysates from TM cells treated with vehicle-control (ctrl) or 1 mM 4MU for 3 and 4 days. All media lanes are from different lanes of the same immunoblot. Cell lanes are from adjacent lanes of the same immunoblot. (D) Densitometry of the V1 isoform (265 kDa) in the media showed a significant reduction with 4MU treatment at 3 and 4 days. Versican levels were normalized to total protein loaded and are presented as a percentage of the control. Error bars are the s.e.m.; n = 4 for 3 days, n = 3 for 4 days. * p = 0.031 and ** p = 0.024 by one-way ANOVA. (E) Versican immunostaining of TM cells treated with vehicle-control or 1 mM 4MU for 4 days and viewed by confocal microscopy. Inset shows a negative control with no primary antibodies. Nuclei are stained with DAPI (control and inset) or Draq5 (4MU). Scale bars = 20 µm.

4MU is an inhibitor of HA synthesis. [22], [23], [24] Previously, we found that 4MU treatment decreased HA synthesis by 60–75% in cell lysates and media of porcine TM cells. [25] A cell viability assay showed no significant difference in the percentage of dead cells between TM cells treated for 5 days with 4MU or vehicle control (Fig. 1A). We then quantitated versican mRNA levels in TM cells treated for 24 hours with various doses of 4MU (0 to 2 mM) using qRT-PCR. There was a significant dose-dependent decrease in versican mRNA levels in response to 4MU treatment (Fig. 1B). Western immunoblots were performed to detect versican protein in the media and lysates of TM cells treated for 3 or 4 days with 1 mM 4MU (Fig. 1C). The V1 isoform was the most abundant protein isoform detected (265 kDa), while smaller amounts of V0 (372 kDa) and V2 (182 kDa) were also found. The V3 (74 kDa) isoform was not detected. This data is consistent with the relative levels of isoform-specific transcripts detected by qRT-PCR in TM cells. [8], [27] Densitometry was used to quantify the intensity of the V1 isoform bands in the media immunoblot. With 4MU treatment, versican V1 protein levels in the media were reduced approximately 40 and 60% at 3 and 4 days, respectively, compared to vehicle-treated control cells (Fig. 1D) when accounting for differences in total protein concentration of each sample. Treatment with 4MU did not affect the ratio of versican isoforms (data not shown). Versican was also reduced in 4MU-treated cell lysates by immunoblotting (Fig. 1C) and in the ECM of 4MU-treated TM cells by immunofluorescence and confocal microscopy (Fig. 1E). Assembly of versican into fibrils was virtually eliminated after 4 days of treatment.

### Effects of 4MU on Fibronectin

**Figure 2:**
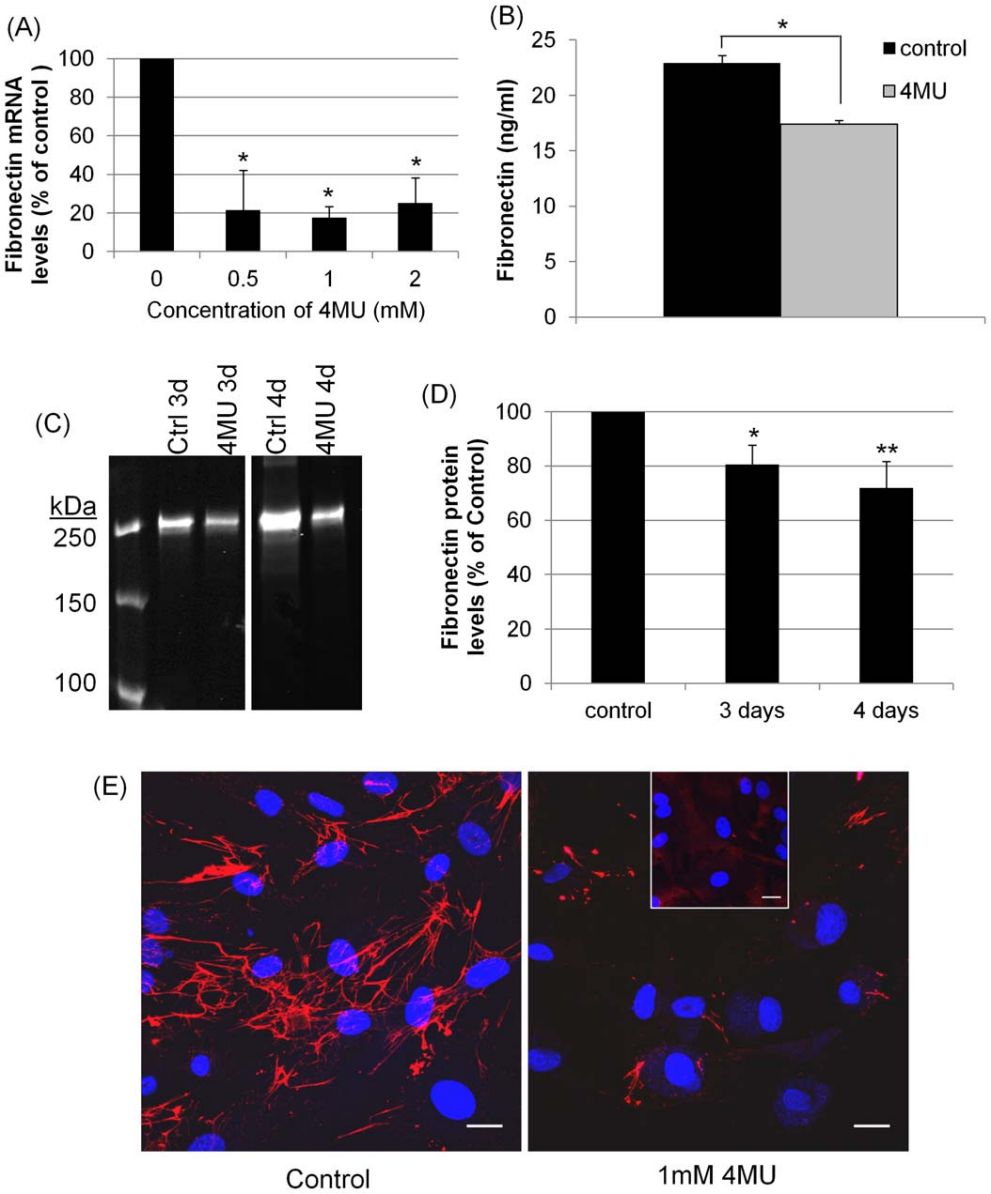
Fibronectin levels in response to 4MU treatment. (A) Fibronectin mRNA levels by qRT-PCR in TM cells treated with 0, 0.5, 1 and 2 mM 4MU for 36 hours. mRNA levels were normalized to 18S RNA levels and presented as a percentage of the control. Error bars are the s.e.m.; n = 3; * P<0.01 by one-way ANOVA. (B) Fibronectin ELISA assay to measure total fibronectin (RIPA lysates and media) protein levels in TM cells. Cells were treated for 4 days with 1 mM 4MU or vehicle-control. Error bars are the s.e.m.; n = 3. * P = 0.0001 by an unpaired Student’s t-test. (C) Western immunoblots of fibronectin protein in conditioned media from TM cells treated with vehicle-control (ctrl) or 1 mM 4MU for 3 and 4 days. The MW markers and 3 day samples are from adjacent lanes of one immunoblot and the 4 days lanes are from adjacent lanes of a different immunoblot. (D) Densitometry of fibronectin showed a significant reduction with 4MU treatment. Fibronectin levels were normalized to total protein loaded and are presented as a percentage of the control. Error bars are the s.e.m.; n = 6 for 3 days, n = 5 for 4 days. * p = 0.039 and ** p = 0.02 by one-way ANOVA. (E) Fibronectin immunostaining of TM cells treated with vehicle-control or 1 mM 4MU for 4 days and viewed by confocal microscopy. Nuclei are stained with DAPI (control) or Draq5 (4MU). Scale bars = 20 µm.

Next, we investigated the effects of 4MU on fibronectin mRNA and protein levels. After 36 hours of treatment, fibronectin mRNA levels were significantly reduced by all concentrations of 4MU tested (Fig. 2A). A fibronectin ELISA assay was used to quantitate protein levels in 4MU-treated TM cells. At 4 days, there was a significant decrease in total fibronectin (RIPA + media) synthesized in 4MU-treated TM cells compared to vehicle-treated control cells (Fig. 2B). Fibronectin protein levels in media were compared by Western immunoblotting and densitometry. A significant reduction in fibronectin protein was observed after 3 and 4 days of treatment compared to vehicle-treated control (Fig. 2C, D). Fibronectin was also reduced in the ECM of 4MU-treated TM cells by immunofluorescence and confocal microscopy (Fig. 2E). In 4MU-treated TM cells, the amount of fibronectin fibrils was severely reduced, especially the amount of thin fibrils.

### HAS Gene Silencing Effects on Versican and Fibronectin

**Figure 3:**
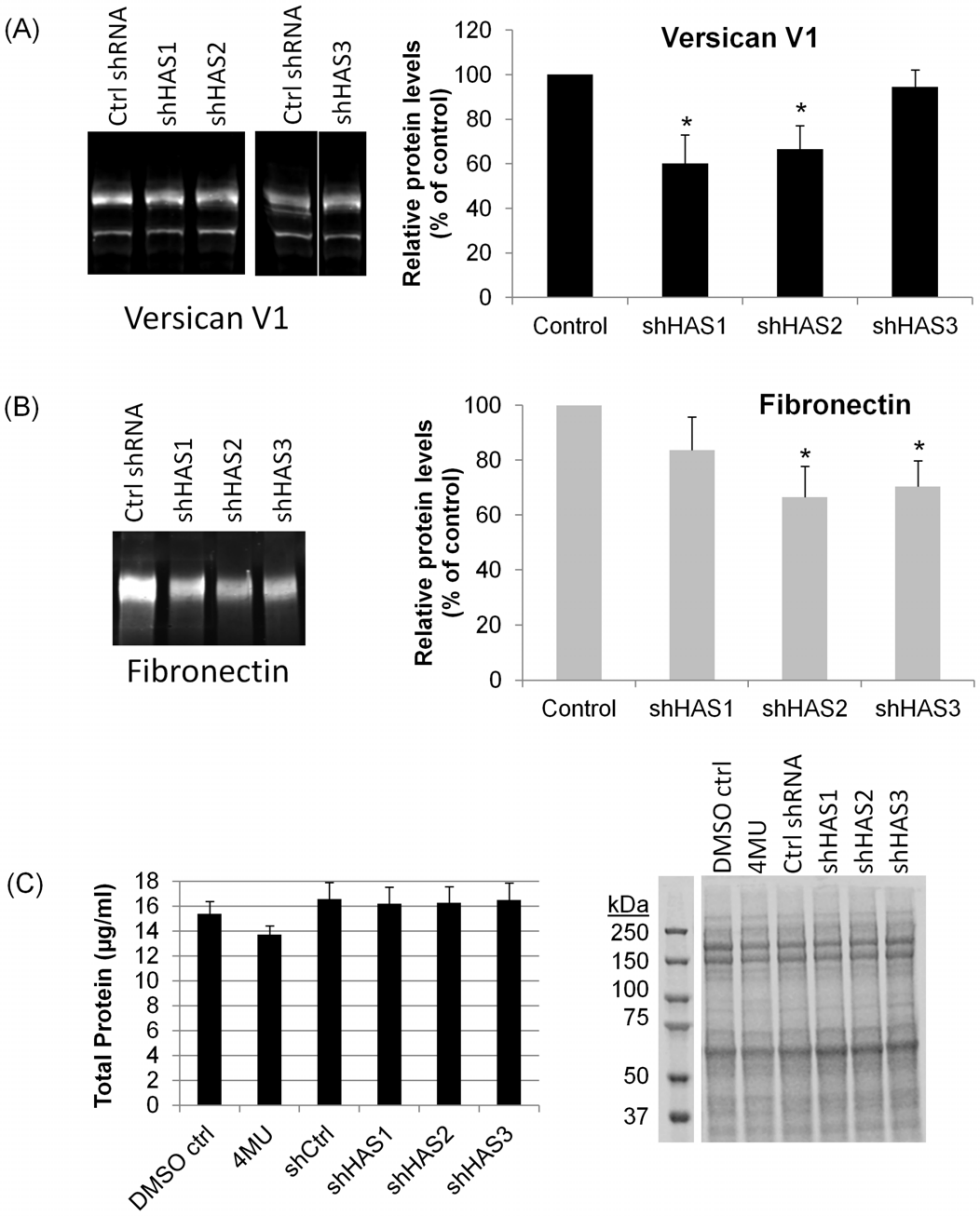
Versican and fibronectin protein levels in response to HAS gene silencing. TM cells were infected with shHAS silencing lentivirus targeting individual HAS genes. Versican and fibronectin protein levels in conditioned serum-free media were assessed by Western immunoblot on 7.5% (versican) or 5% (fibronectin) SDS-PAGE gels. (A) Versican V1 in shHAS-infected TM cells. Images originate from two separate experiments: ctrl shRNA, shHAS1 and shHAS2 were from adjacent lanes on one immunoblot, while ctrl shRNA and shHAS3 were from non-adjacent lanes of a second immunoblot. Densitometry of the versican V1 isoform showed a significant reduction in protein levels following shHAS1 and shHAS2 lentivirus infection (n = 4). (B) Fibronectin in shHAS-infected TM cells. Densitometry of fibronectin showed a significant reduction in protein levels following shHAS2 and shHAS3 lentivirus infection (n = 3). The fibronectin immunoblot shows adjacent lanes from a single blot. Versican and fibronectin values were normalized to total protein loaded and are presented as a percentage of the control. Error bars are the s.e.m. * P<0.04 by one-way ANOVA. (C) Total protein (RIPA + media) was quantitated using the BCA assay for vehicle control (n = 7), 4MU-treated (4 day treatment; n = 7), shControl (n = 6) and shHAS silenced TM cells (n = 6). No significant differences were observed by ANOVA. A representative Coomassie-stained gel (7.5%) shows approximately equal loading of lanes for all treatments.

4MU inhibits all three HAS synthases and depletes the cellular concentration of UDP-GlcUA, a building block of HA. [22], [23], [36] Previously, we generated silencing lentivirus to knockdown expression of each individual HAS gene. [25] Knockdown reduced HAS mRNA levels by 70–98%, HAS protein levels by 25–35% and the amount of HA synthesized by approximately 25–50% as quantitated by an ELISA assay. [25] In order to assess whether HAS gene silencing also reduces versican and fibronectin protein levels, we infected TM cells with each of the HAS silencing lentiviruses (shHAS1, shHAS2 and shHAS3). Consistent with the results of the 4MU treatment, we found reductions in versican and fibronectin protein levels in the media following infection with shHAS silencing lentivirus (Fig. 3). These reductions were also found in RIPA lysates (not shown). Although all 3 shHAS reduced versican and fibronectin levels, versican protein levels were only significantly reduced by shHAS1 and shHAS2 silencing (Fig. 3A), while fibronectin protein levels were only significantly reduced by shHAS2 and shHAS3 silencing (Fig. 3B). Analysis of total protein (RIPA + media) levels by BCA assay showed that 4MU and shHAS silencing did not significantly affect total protein levels produced by TM cells (Fig. 3C). A Coomassie stained gel shows approximately equal loading of lanes.

### Supplementation with Excess UDP-sugar Substrates

**Figure 4:**
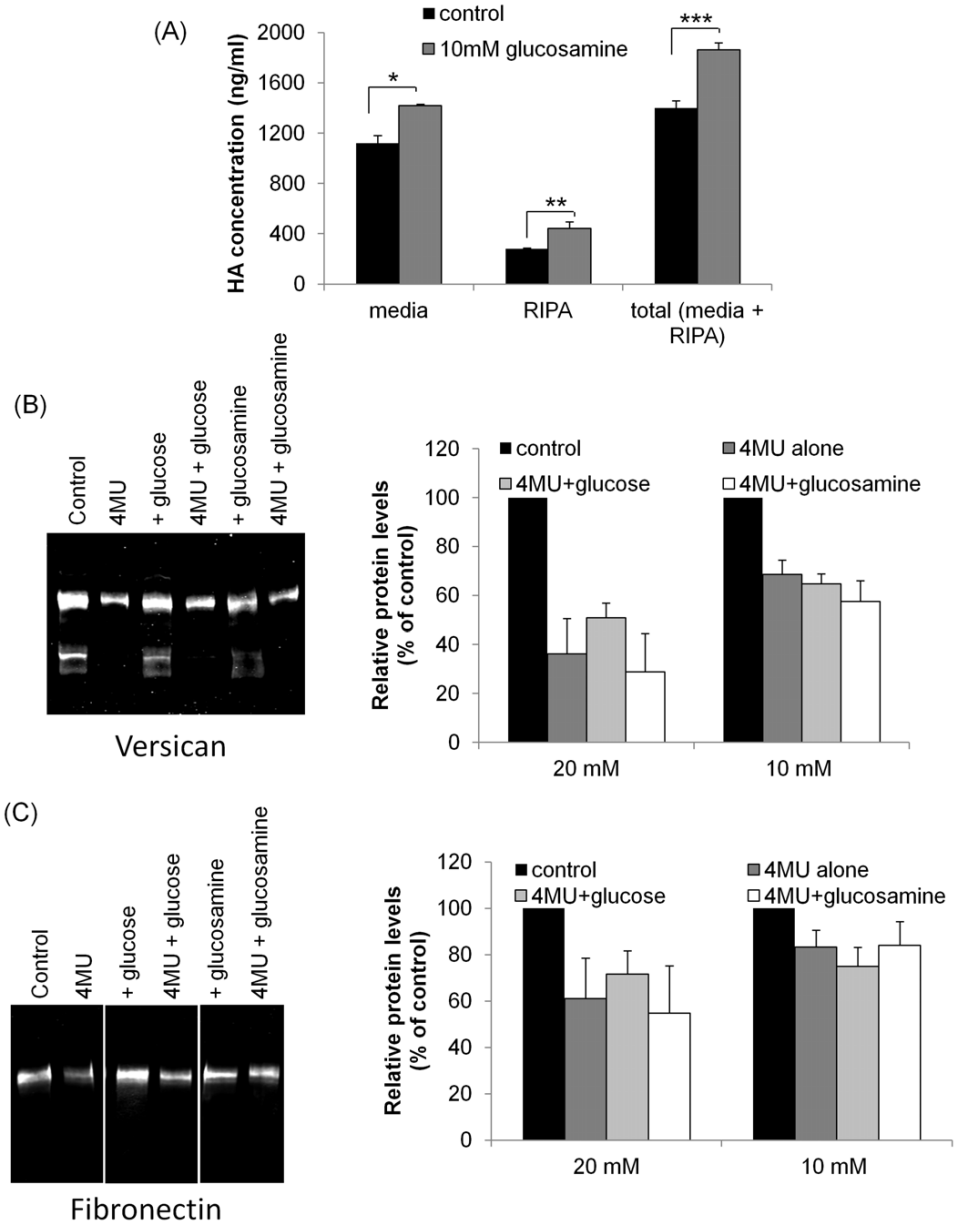
Effect of addition of glucose and glucosamine to 4MU-treated TM cells. (A) An HA ELISA assay was used to quantitate HA levels in TM cells treated with 10 mM glucosamine for 48 hrs. HA levels were significantly increased in media (*p = 0.008), RIPA lysates (**p = 0.04) and total media + RIPA lysates (***p = 0.004) compared to untreated control TM cells. Error bars are the s.e.m; n = 3. (B, C) Confluent TM cells were treated for 4 days with 1 mM 4MU alone, or 4MU with 10 mM glucose or 10 mM glucosamine in serum-free media. Versican and fibronectin protein levels in conditioned media were assessed by Western immunoblot. (B) Versican V1 isoform and (C) fibronectin protein levels were significantly decreased by 4MU treatment. Addition of 20 mM or 10 mM glucose or glucosamine did not significantly alter protein levels compared to 4MU treatment with either glucose or glucosamine supplementation. The Western immunoblots show data from a 10 mM treatment. For the versican immunoblot, all lanes were from adjacent lanes of the same immunoblot. The fibronectin immunoblot shows non-adjacent lanes from the same immunoblot. Versican and fibronectin values were normalized to total protein loaded and are presented as a percentage of the control. Error bars are the s.e.m.; n = 3.

Supplementation of cell culture media with glucosamine was shown to rapidly increase HA synthesis 2.6-fold due to increased cytosolic concentrations of UDP-GlcNAc and UDP-GlcUA. [37] In the same study, glucose did not affect HA synthesis, but it is a precursor of UDP-GlcUA, which 4MU reduces. Equimolar amounts of both UDP-sugar substrates is required for HA synthesis. [17] TM cells are routinely grown in media containing 15.3 mM glucose, which is similar to glucose levels found in aqueous humor (12.2 mM). [38] Therefore, we investigated whether supplementation of media with excess glucose or glucosamine could reverse the effect of 4MU on versican and fibronectin protein levels. First, we analyzed HA levels in TM cells in response to 4MU treatment for 48 hours. HA concentration in media and RIPA cell lysates were shown to significantly increase following treatment with 10 mM glucosamine, as quantitated by an ELISA assay (Fig. 4A). Next, we evaluated versican and fibronectin protein by Western immunoblot and densitometry. Consistent with the data above, both versican and fibronectin levels were significantly reduced by 4MU treatment after 3 days. However, there was no significant restoration of versican (Fig. 4B) or fibronectin (Fig. 4C) levels when the media was supplemented with either 10 mM or 20 mM glucose or glucosamine. By Western immunoblot, glucosamine treatment seemed to decrease the amount of the V2 isoform. Although quantitation of this band showed that this reduction approached significance (p = 0.12) when compared to the v1 band, it was not statistically significant upon analysis of 3 independent experiments.

### Supplementation with Exogenous HA

**Figure 5:**
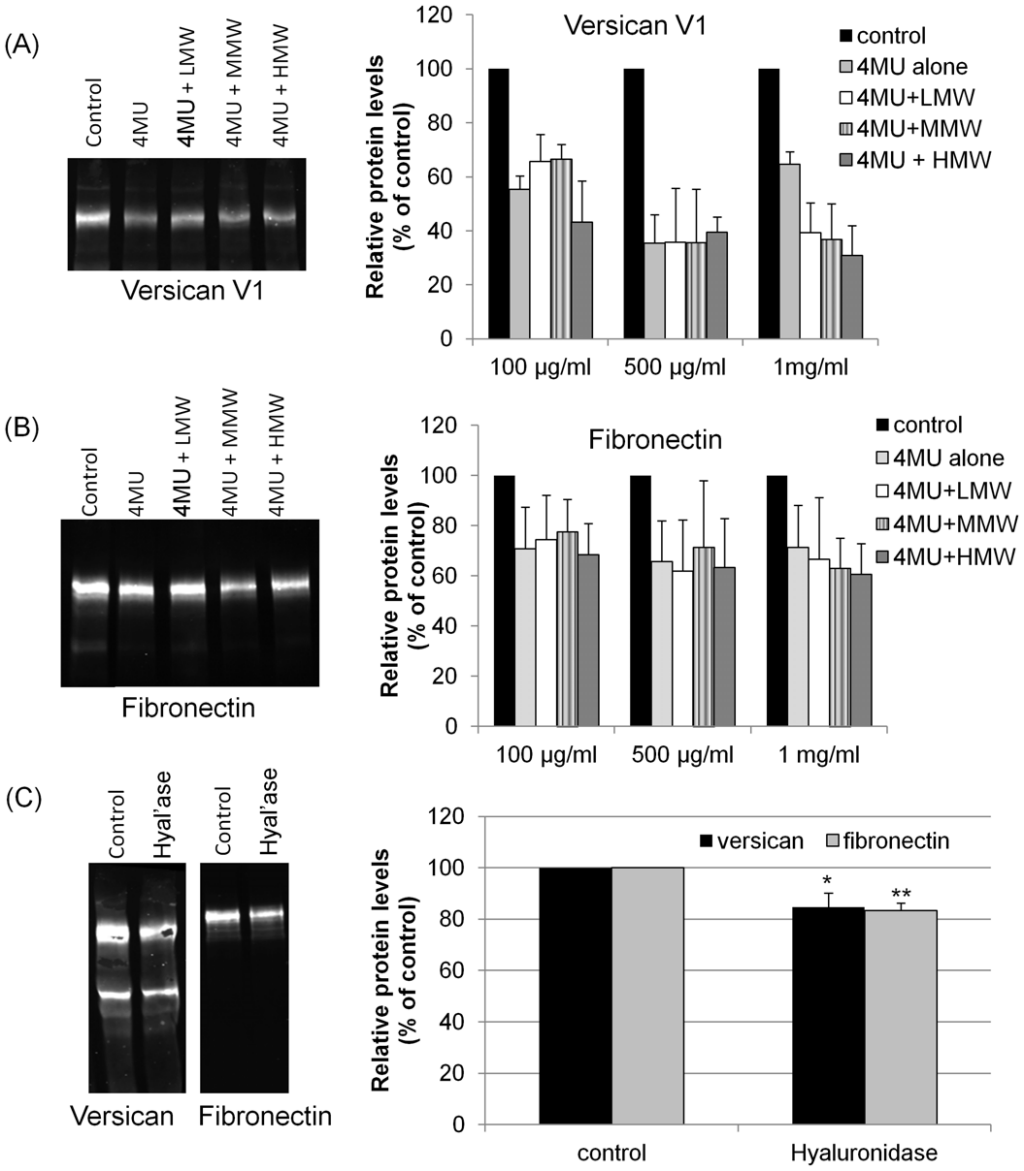
Effect of addition of exogenous HA and hyaluronidase treatment. Confluent TM cells were treated for 2 days with 1 mM 4MU and then HA of different molecular weights (high MW = 1.5 MDa; medium MW = 700 kDa; low MW = 40 kDa) was exogenously added for a further 3 days. Versican and fibronectin protein levels in conditioned media were assessed by Western immunoblot. (A) Versican V1 isoform and (B) fibronectin protein levels are significantly decreased by 4MU treatment in all cases, but addition of 3 concentrations (100 µg/ml, 500 µg/ml and 1 mg/ml) of HA of HMW, MMW and LMW did not significantly alter levels compared to 4MU treatment alone. The Western immunoblots show data from a 500 µg/ml treatment. All lanes were from adjacent lanes of the same immunoblot. Error bars are the s.e.m.; n = 3. (C) Hyaluronidase (1.5 µg/ml; 1 unit) was added to confluent TM cells in serum-free media each day for 3 days. Western immunoblots and densitometry showed that versican V1 and fibronectin protein levels were significantly decreased by hyaluronidase treatment. All lanes were from adjacent lanes of a single immunoblot. Versican and fibronectin values were normalized to total protein loaded and are presented as a percentage of the control. Error bars are the s.e.m.; n = 4; * p = 0.03 and ** p = 0.001 by one-way ANOVA.

Next, we asked whether addition of exogenous HA could reverse the effect of 4MU on versican and fibronectin protein levels. The effects of HA of different sizes were investigated: high MW (1.5 MDa), medium MW (700 kDa) and low MW (40 kDa) HA. TM cells were treated with 4MU for 2 days prior to the addition of exogenous HA for a further 3 days (Fig. 5). Versican and fibronectin protein levels were again significantly reduced by 4MU treatment alone. Western immunoblots showed there was no significant difference in levels when 500 µg/ml HA of any size was added exogenously (Fig. 5A, B). Densitometry of immunoblots showed that similar results were obtained when 100 µg/ml and 1 mg/ml exogenous HA was added to 4MU-treated TM cells. We then investigated whether treatment of TM cells with hyaluronidase, to degrade endogenous HA, affected versican and fibronectin protein levels (Fig. 5C). Both versican V1 and fibronectin protein levels were significantly decreased upon daily hyaluronidase treatment for 3 days, as shown by Western immunoblot and densitometry.

### Immunolocalization of HA, Versican and Fibronectin in 4MU-treated TM Tissue

**Figure 6:**
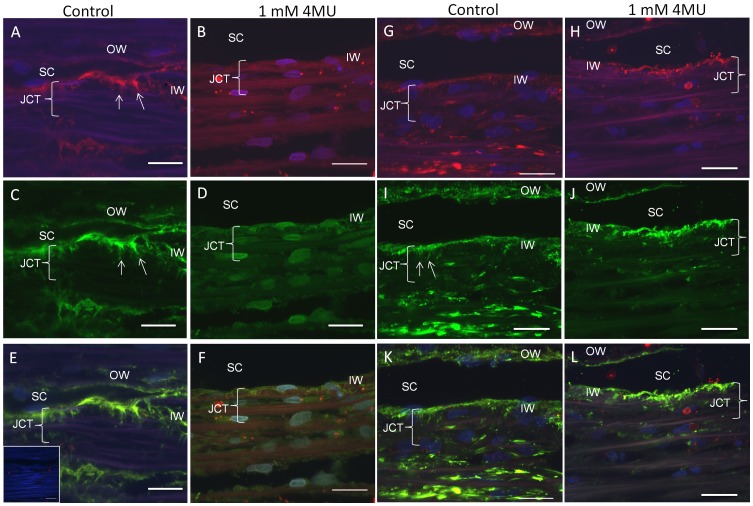
Immunostaining of 4MU-perfused trabecular meshwork tissue. Human anterior segments were perfused with vehicle (A, C, E, G, I, K) or 1 mM 4MU (B, D, F, H, J, L) continuously for 5 days. Frontal sections were cut and stained with bHAbp (A, B, G, H), versican (C, D) or fibronectin (I, J) antibodies. Colocalization of HAbp and versican (E, F) and HAbp and fibronectin (K, L) are also shown. Arrows point to pillar-like staining that runs perpendicular to Schlemm’s canal. Confocal acquisition settings were identical between control and 4MU-treated eyes. Inset in E shows negative control. SC = Schlemm’s canal; OW = outer wall; JCT = juxtacanalicular region; IW = inner wall. Scale bars = 20 µm.

Previously, we showed that perfusion of anterior segments with 4MU significantly reduced outflow facility in human eyes. [25] In this study, we evaluated the localization patterns of HA-binding protein (HAbp), versican and fibronectin in control and 4MU-treated TM tissue (Fig. 6). HA, assessed by HAbp binding, was found in the juxtacanalicular (JCT) region of the TM, surrounding some outer beams of the TM tissue and in the outer wall of Schlemm’s canal of control eyes (Fig. 6A, G). While HAbp staining was somewhat variable, as we have observed previously, [25], [39] perfusion of 4MU resulted in a large decrease in the amount of HA detected in the outer beams and inner wall, while the pattern of staining in the JCT was reduced and became more punctate (Fig. 6B, H). Versican immunostaining in control eyes was abundant in the JCT region, with less staining in the outer beams and in the outer wall of Schlemm’s canal (Fig. 6C). Versican immunostaining was in pillar-like structures that run perpendicular to Schlemm’s canal, a pattern similar to that observed previously. [28] HAbp staining showed good colocalization with versican in control TM (Fig. 6E). In 4MU-treated tissue, overall levels of versican immunostaining were reduced and the staining pattern was altered, especially in the JCT region (Fig. 6D). In these eyes, punctate HAbp staining did not colocalize with versican immunostaining (Fig. 6F). Fibronectin staining in control eyes was abundant (Fig. 6I). Immunostaining was observed in the inner wall and JCT region, with pillars of fibronectin staining running perpendicular to Schlemm’s canal. Fibronectin also stained the outer TM beams and the outer wall of Schlemm’s canal. Fibronectin immunostaining partially colocalized with HAbp staining (Fig. 6K). In 4MU-treated eyes, fibronectin staining was highly reduced in the outer beams and outer wall, while the pattern of staining was more punctate in the JCT region and there was partial loss of the pillar-like staining (Fig. 6J, L).

### Effects of 4MU on Selected ECM Genes

**Figure 7:**
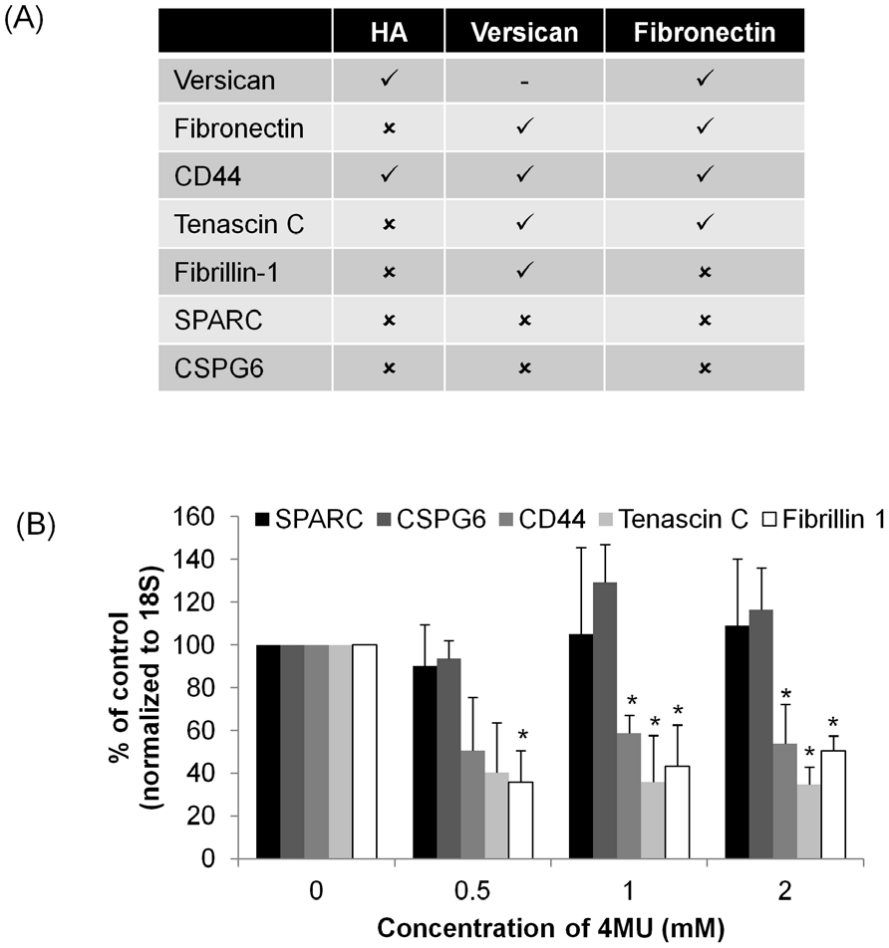
The effects of 4MU on mRNA levels of other ECM genes. (A) The table summarizes the previously identified binding interactions of HA, versican and fibronectin reported in the literature. (B) Quantitative RT-PCR of mRNA levels of CD44, tenascin C, fibrillin-1, CSPG6 and SPARC in TM cells treated with 0, 0.5, 1 and 2 mM 4MU for 36 hours. All mRNA levels were normalized to 18S RNA levels and presented as a percentage of the control. Error bars are the s.e.m. N = 3, apart from CSPG6, where n = 4. * P<0.05 by one-way ANOVA.

The effect of 4MU on mRNA levels of other ECM genes expressed by TM cells was investigated by qRT-PCR (Fig. 7). We focused on CD44, tenascin C and fibrillin-1, since these proteins interact with HA, versican and/or fibronectin (Fig. 7A). [40], [41], [42], [43] In addition, we investigated bamacan (CSPG6), a CS-bearing proteoglycan that does not have a HA-binding link domain, [44] and SPARC, a matricellular protein that is highly expressed in the TM and enhances fibronectin assembly. [45], [46] There are no reports in the literature of either SPARC or CSPG6 interacting with HA, versican or fibronectin. The mRNA levels of CD44, tenascin C and fibrillin-1 were all significantly reduced by treatment with 4MU (Fig. 7B). However, mRNA levels of CSPG6 and SPARC were not significantly affected by any concentration of 4MU tested.

### Effect of Versican Silencing on HA Levels

**Figure 8:**
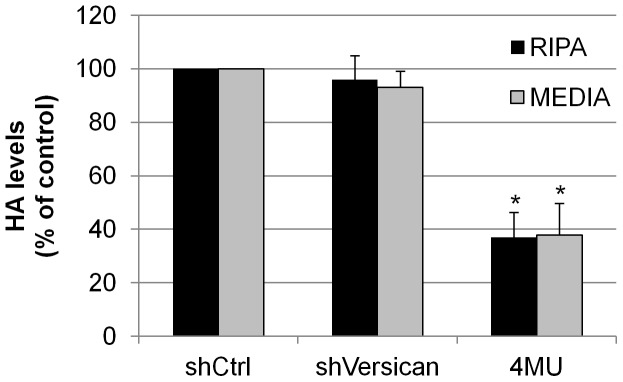
HA levels quantitated by a competitive HA ELISA assay. TM cells were infected with versican silencing lentivirus (shVersican) for 72 hours and then changed to serum-free media for a further 24 hours. 4MU (1 mM) was added to confluent TM cells for 24 hours. HA concentration in RIPA lysates and serum-free media was quantitated using a competitive HA ELISA assay. Error bars are the s.e.m. N = 8 for shVersican and n = 7 for 4MU. * P<0.0001 by one-way ANOVA.

Since HA and versican expression may be linked, [33] we also evaluated whether a reduction of versican affected HA concentration. shVersican silencing lentivirus, which was previously described, [28] was added to TM cells in culture. HA levels in the RIPA lysates and media were quantified using an HA ELISA assay. As a positive control, we also quantified levels in 4MU-treated TM cells. Versican silencing did not significantly affect HA levels in TM cells, while 4MU treatment highly reduced HA concentrations in both the RIPA lysates and media (Fig. 8).

## Discussion

Versican and fibronectin levels were reduced in response to three different methods of decreasing HA levels: 4MU treatment, shHAS gene silencing, and hyaluronidase treatment. This implies that versican and fibronectin protein reductions are due to decreased HA concentration rather than a direct effect of the treatments on versican and fibronectin. Moreover, the 4MU effect must be on the HAS enzymes since increasing UDP-sugar substrate concentration did not restore versican and fibronectin levels. Also, only *de novo* synthesized HA was able to alter versican and fibronectin levels since addition of exogenous HA of various sizes did not substantially reverse the 4MU effect.

The forms of HA produced by different HASs appear to be important since silencing HAS3 did not significantly affect versican protein levels, while knockdown of HAS1 had no significant effect on fibronectin protein levels. This suggests that the abundance and size of HA chains may modulate the effect since HAS1 synthesizes low amounts of high molecular weight HA chains, while HAS3 generates abundant short HA chains. [18], [19] Although we did not measure HA size in this study, our data suggests that HA chains produced by HAS1 and HAS2 are required to maintain versican expression, while HA abundance may be more important in maintaining fibronectin expression. However, silencing of each individual HAS reduced HA levels in PTM cells approximately 40% for shHAS1 and shHAS2, but only 20% for shHAS3. [25] Therefore, the relative efficacy of shHAS3 knockdown on HA levels was reduced, possibly masking an effect of shHAS3 on versican levels.

HA is a major constituent of the pericellular matrix and, conceptually, HA is ideally placed to act as a central organizer of ECM molecules. The molecular composition of the pericellular matrix is tightly regulated and alterations in the levels of one component will have profound consequences on ECM structural organization and matrix architecture during dynamic ECM turnover events. HA has been proposed to aid retention of newly synthesized ECM molecules prior to assembly by either binding HA directly, or via indirect associations with other matrix molecules bound to HA. [14], [16] Our data support this hypothesis since we observed a large reduction in versican and fibronectin fibrils in 4MU-treated TM cells. A critical concentration of versican and fibronectin molecules is required before fibril assembly can occur. [47], [48] Thus, a reduction in overall amounts and/or disruption in retention of these molecules by HA would likely alter or disrupt fibril assembly. Changes in fibronectin assembly could be particularly catastrophic since fibronectin is thought to orchestrate the assembly of other ECM components. It would also reduce the mechanical support for cell attachment. In this regard, a recent study showed the number and size of filopodia of human esophageal squamous carcinoma cells were reduced upon HAS2 and HAS3 gene silencing, which may be partially explained by a reduction in fibronectin levels. [49].

In this study, versican knockdown by silencing did not affect total HA concentration as measured by a HA ELISA assay. Conversely, diminished versican deposition in a transgenic mouse strain reduced the amount of HA in the pericellular matrix and increased the amount of free HA fragments. [47] The differences between this study and ours are likely due to systems and conditions. HA length was not measured in this study so there remains the possibility that HA fragments may have been generated in shVersican-infected TM cells.

A primary function of TM cells in the eye is to maintain IOP homeostasis by regulating the outflow resistance. [4], [5] Previously, we have shown that RNAi silencing of HAS1 or HAS2, and reduction of HA levels by 4MU increased outflow resistance in human eyes. [25] The results presented here suggest that some of the effects on resistance caused by reduced HA levels may be due to decreased versican synthesis, since versican knockdown also increases outflow resistance. [28] Certainly versican immunostaining in 4MU-treated TM tissue was reduced and the immunostaining pattern was altered. Loss of HA and/or versican may disrupt open flow channels in the TM leading to the observed increase in outflow resistance. Previous studies noted that HA levels are reduced in normal aged trabecular meshwork and are highly reduced in POAG eyes. [21], [50] Therefore, it is plausible that as aging progresses, slight reductions of versican and fibronectin may be tolerated in normal eyes, but larger changes in HA concentration may decrease versican and fibronectin levels to such an extent that a tipping point is reached. Consequently, ECM organization may be disrupted and the TM may no longer be able to respond correctly in order to homeostatically adjust IOP. We speculate that this is a gradual process taking many decades to produce and could contribute to the pathogenesis of POAG.

After synthesis, HA can be retained at the cell surface due to extended interactions with its synthases, or by binding to cell surface receptors such as CD44 and RHAMM. [16] These receptors readily respond to changes in amounts and size of cell surface HA, and transmit signals intracellularly. This form of signaling seems like a good candidate for linking HA levels with versican and fibronectin synthesis. The apparent co-regulation of five ECM proteins with overlapping binding domains suggests potential shared transcriptional regulation. A recent microarray study showed that 4MU treatment altered levels of many genes, but the mechanism was not established. [51] Other studies have reported changes in CD44 and decorin levels in response to HAS gene silencing or 4MU treatment. [52], [53], [54], [55] For instance, total CD44 mRNA expression was reduced in HAS2 siRNA-treated human breast cancer cells, while a different study reported that certain CD44 alternative transcripts were down-regulated in HAS1 siRNA-treated bladder cancer cells. [52], [54] Conversely, there was no effect of 4MU treatment on CD44 protein levels in T-cells. [55] In TM cells, we showed that 4MU treatment decreased total CD44, tenascin C and fibrillin-1 mRNA levels. Combined, these observations suggest that the effects of HA reduction on genes may be cell-type and/or cell niche dependent.

In summary, we have shown that a decrease in HA concentration in TM cells profoundly affects synthesis of certain ECM molecules including versican and fibronectin. The results provide additional evidence that HA is critical for maintaining the correct balance and structural organization of other extracellular components. Coordinated coupling of HA synthesis with that of other ECM proteins should facilitate aqueous humor outflow and IOP homeostasis in the eye.

## Methods

### Trabecular Meshwork Primary Cell Culture

Porcine eyes were acquired from the local slaughterhouse (Carlton Farms, Carlton, OR). Primary TM cells were isolated from TMs dissected from porcine eyes as previously described. [56], [57] TM cells were cultured in medium glucose (15.3 mM) Dulbecco’s Modified Eagle’s Medium (DMEM; Invitrogen, Carlsbad, CA) containing 10% fetal bovine serum (FBS) and 1% penicillin-streptomycin-gentamicin. Primary TM cells were used for a maximum of 5 passages. For 4MU treatments, confluent TM cells were washed with phosphate-buffered saline (PBS), made serum-free and 4-methylumbelliferone (Sigma, St Louis, MO) dissolved in DMSO was added at the stated concentrations and times. DMSO alone was used as a vehicle control. Cell viability was assessed using the live-dead viability kit (Invitrogen). TM cells were treated for 5 days with 1 mM 4MU or vehicle control and stained following the manufacturer’s instructions. Stained cells were viewed by confocal microscopy and the number of live and dead cells was counted in 6 random microscope fields. The number of dead cells per field was expressed as a percentage of total cells, percentages were averaged and significance was determined.

For HAS and versican silencing experiments, short hairpin RNA (shRNA) silencing lentivirus (10^6^ pfus) was added with 6 µg/ml polybrene (Sigma) at the time of plating in serum-containing medium. TM cells were grown for 3 days to allow infection and silencing, then washed with PBS and made serum-free for the times stated. The methods and efficacy of shHAS and shVersican gene silencing has been described previously. [25], [28] For sugar supplementation experiments, an additional 10 mM or 20 mM D-glucose (Sigma), or 10 mM or 20 mM glucosamine (Sigma), was added to serum-free medium of confluent TM cells in the presence of 1 mM 4MU for 3 days. For exogenous HA treatments, HA of known sizes was purchased from Lifecore Biomedical, Chaska, MN. Confluent TM cells were made serum-free and treated with 1 mM 4MU for 2 days prior to addition of 100, 500 µg/ml or 1 mg/ml of high MW HA (HMW; 1.5 MDa), medium MW HA (MMW; 700 kDa) or low MW HA (LMW; 40 kDa) for a further 3 days. For hyaluronidase treatments, hyaluronidase (1 unit in PBS; from *Streptomyces hyalurolyticus;* MP Biomedicals, Solon, OH) was added to confluent TM cells in serum-free media each day for 3 days in the presence of protease inhibitor cocktail for tissue culture (contains aprotinin, bestatin, E-64, leupeptin and pepstatin A; Sigma).

### Quantitative RT-PCR

Total RNA was isolated from 4MU-treated or vehicle control-treated TM cells using cells-to-cDNA lysis buffer (Ambion, Austin, TX) and cDNA was generated using Superscript III reverse transcriptase (Invitrogen, Carlsbad, CA). Quantitative RT-PCR (qRT-PCR) was used to evaluate mRNA levels using specific primers for versican, fibronectin, CD44, CSPG6 (bamacan), SPARC, tenascin C and fibrillin-1 (Integrated DNA Technologies, Inc, San Diego, CA) and methods described previously.^33, 34^ Results were normalized to 18S RNA, which acted as a housekeeping gene, and expressed as a percentage of vehicle control-treated TM cells.

### Western Immunoblotting

RIPA lysates and media were harvested from confluent TM cells treated with 1 mM 4MU for either 3 or 4 days. For versican, 1 ml of serum-free media was acid-precipitated and the pellet was washed and resuspended in 1× SDS-PAGE sample buffer. For fibronectin, 30 µl of media was loaded. Proteins were separated on 5% or 7.5% (Fig. 3A, C) SDS-PAGE gels (BioRad Labs, Hercules, CA) under reducing conditions and transferred to nitrocellulose. Some gels were stained with Coomassie blue. Immunoblots were probed with mouse anti-versican antibody (Developmental studies hybridoma bank, University of Iowa, Iowa City, IA) or a rabbit fibronectin polyclonal antibody (Abcam, Cambridge, MA). Secondary antibodies were IRDye 700–conjugated anti-rabbit and IRDye 800-conjugated anti-mouse (Rockland Immunochemicals, Gilbertsville, PA). Immunoblots were imaged using the Odyssey infrared imaging system (Li-cor Biosciences, Lincoln, NE). Gel bands were quantitated using FIJI software (http://fiji.sc/wiki/index.php/Fiji) following background correction. Average pixel intensity was corrected for differences in total protein concentration in each sample as determined by a BCA assay (Pierce, Rockford, IL).

### Immunofluorescence and Confocal Microscopy

TM cells were grown on collagen type I-coated Bioflex membranes (FlexCell Int., Hillsborough, NC) as described previously. [29] Confluent TM cells were treated with 1 mM 4MU or DMSO vehicle control for 4 days. At the end of the experiment, cells were fixed with 4% paraformaldehyde, membranes were removed from the dish, placed on a glass slide and blocked with CAS block (Invitrogen). Membranes were then immunostained with a versican monoclonal antibody and Alexa-fluor 488-conjugated secondary antibody or a fibronectin polyclonal antibody and Alexa-fluor 594-conjugated secondary antibody. Coverslips were mounted with ProLong gold containing DAPI nuclear stain (Invitrogen), or nuclei were stained with Draq5 (Abcam) and mounted in Fluoromount G (Southern Biotech, Birmingham, AL). Images were obtained with a Fluoview FV1000 laser confocal scanning microscope (Olympus, San Diego, CA) and processed with FIJI software.

Immunostaining was also performed on human anterior segments from donor eyes acquired from Lions Eyebank of Oregon (Portland, OR). Use of donor eye tissue was approved by Oregon Health & Science University Institutional Review Board and experiments were conducted in accordance with the tenets of the Declaration of Helsinki for the use of human tissue. Average age ± s.e.m. of donor eyes was 84.16±2.12 years, range, 78–92 years, n = 6. Anterior segments were perfused continuously with 1 mM 4MU or vehicle control for 5 days as described previously. [25] At the end of perfusion, TM tissue was immersion-fixed in 4% paraformaldehyde and frontal sections were cut. [28], [58] Tissues were blocked and incubated overnight at 4°C with a versican polyclonal antibody (Novus Biologicals, Littleton, CO) or the fibronectin polyclonal antibody paired with biotinylated HAbp (EMD Biosciences, San Diego, CA). Primary antibodies were detected with Alexa-fluor 488-conjugated anti-rabbit and Alexa-fluor 594-conjugated Streptavidin, respectively. Sections were immersed in Slowfade Gold Antifade with DAPI (Invitrogen) on glass slides and imaged by confocal microscopy. The length of time that the tissue was in antifade led to slight variability in the level of nuclear staining between images. Confocal image acquisition settings and number of sections stacked for each image were maintained between control and 4MU-treated eyes. Images show representative images from 6 eyes examined.

### ELISA Assays

A human fibronectin sandwich ELISA kit (Syd Labs, Malden, MA) was used to quantitate fibronectin protein levels in TM cells treated with 4MU for 4 days. Three independent samples were collected for each treatment. Serum-free media was harvested, diluted 1∶1 with dilution buffer supplied with the kit and protease inhibitor cocktail (Sigma) was added. Adherent TM cells were washed with PBS and cells/ECM was extracted in RIPA buffer containing 2M urea to aid solubilization of cellular fibronectin and protease inhibitor cocktail. RIPA lysates were diluted 1∶6 with dilution buffer. Fibronectin standard solutions (0 to 10 ng/ml) were prepared as described by the manufacturer. Diluted samples and standards (100 µl) were added in triplicate to each well of a 96-well plate. The ELISA assay procedure was performed following the manufacturer’s protocol. A standard curve was generated from which the fibronectin concentration in each sample was determined. These values were then averaged, multiplied by the dilution factor and values from media and RIPA lysates were totaled to obtain the total fibronectin concentration in each sample. An average fibronectin concentration from 3 independent samples was then calculated.

To quantitate HA levels in TM cells, a competitive HA ELISA assay (Echelon Bioscience, Salt Lake City, UT) was performed. For glucosamine experiments, confluent TM cells were treated with 10 mM glucosamine in serum-free media for 48 hours. For shHAS knockdown experiments, TM cells were infected with 10^6^ pfus of shVersican lentivirus or shControl for 72 hours. The infected cells were washed in PBS and serum-free DMEM was added for a further 24 hrs. As a positive control, confluent TM cells were treated with 1 mM 4MU for 24 hours in serum-free DMEM. For all treatments, media was harvested and the cells and ECM were scraped into RIPA buffer on ice. HA levels in media and RIPA lysates were quantitated following the manufacturer’s instructions. HA levels in ng/ml were calculated from a standard HA curve run on each plate. Each sample was tested in triplicate, individual results were averaged and a percentage of the control was calculated.

### Statistical Analysis

At least 3 independent experiments were performed for each experiment and data are presented as the mean ± the standard error of the mean (s.e.m.). Significance (P<0.05) was determined using one-way analysis of variance (ANOVA) or an unpaired Student’s t-test.
